# Oncogenic PAX6 elicits CDK4/6 inhibitor resistance by epigenetically inactivating the LATS2‐Hippo signaling pathway

**DOI:** 10.1002/ctm2.503

**Published:** 2021-08-23

**Authors:** Yi Zhang, Long‐Jun He, Lin‐Lin Huang, Sheng Yao, Nan Lin, Ping Li, Hui‐Wen Xu, Xi‐Wen Wu, Jian‐Liang Xu, Yi Lu, Yan‐Jie Li, Sen‐Lin Zhu

**Affiliations:** ^1^ Department of Gastroenterology and Hepatology, the First Affiliated Hospital Sun Yat‐sen University No.58 Zhongshan 2nd Road Guangzhou 510630 China; ^2^ Department of Hepatobiliary surgery, the Third Affiliated Hospital Sun Yat‐sen University No.600 Tian he Road Guangzhou 510630 China; ^3^ Department of Hepatic surgery, the First Affiliated Hospital Sun Yat‐sen University No.58 Zhongshan 2nd Road Guangzhou 510080 China; ^4^ State Key Laboratory of Oncology in South China Cancer Center Sun Yat‐sen University No.651 Dongfeng Road East Guangzhou 510060 China; ^5^ Department of Gastroenterology and Hepatology Guangdong Provincial People's Hospital/Guangdong Academy of Medical Sciences No.106 Zhongshan 2nd Road Guangzhou 510080 China

**Keywords:** CDK4/6 inhibitor, DNA methylation, Hippo signaling pathway, PAX6

## Abstract

Intrinsic resistance to CDK4/6 inhibitors hinders their clinical utility in cancer treatment. Furthermore, the predictive markers of CDK4/6 inhibitors in gastric cancer (GC) remain incompletely described. Here, we found that PAX6 expression was negatively correlated with the response to palbociclib *in vitro* and *in vivo* in GC. We observed that the PAX6 expression level was negatively correlated with the overall survival of GC patients and further showed that PAX6 can promote GC cell proliferation and the cell cycle. The cell cycle is regulated by the interaction of cyclins with their partner serine/threonine cyclin‐dependent kinases (CDKs), and the G1/S‐phase transition is the main target of CDK4/6 inhibitors. Therefore, we tested whether PAX6 expression was correlated with the GC response to palbociclib. We found that PAX6 hypermethylates the promoter of *LATS2* and inactivates the Hippo pathway, which upregulates cyclin D1 (CCND1) expression. This results in a suppressed response to palbociclib in GC. Furthermore, we found that the induction of the Hippo signaling pathway or treatment with a DNA methylation inhibitor could overcome PAX6‐induced palbociclib resistance in GC. These findings uncover a tumor promoter function of PAX6 in GC and establish overexpressed PAX6 as a mechanism of resistance to palbociclib.

## Graphical abstract

PAX6 can promote gastric cancer progression. PAX6 can promote palbociclib in gastric cancer via suppressing Hippo signaling. Induction of the Hippo signaling pathway or treatment with DNA methylation inhibitor could overcome PAX6‐induced palbociclib resistance in gastric cancer.

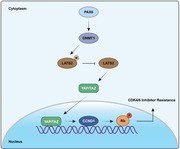

## 1 INTRODUCTION

Gastric cancer (GC) is an aggressive malignancy with high mortality rates, and most patients with advanced GC are unresponsive to existing chemotherapy.^1^ Therefore, a large number of patients with GC are resistant to standard treatments and require new therapeutic methods. Targeting cyclin‐dependent kinases (CDKs) have emerged as an alternative method to treat cancer, as they are fundamental drivers of the cell cycle, as well as initiators and progressors of various malignancies.^2^ CDK4/6 inhibitors (CDK4/6i) are effective in treating several cancers, such as breast cancer and melanoma.^3^ Recently, resistance to CDK4/6i has been considered a near‐inevitability in most malignancies, including those with GC.^4^ The underlying mechanisms of resistance to CDK4/6i are multivariate, and research is ongoing. Biomarkers for identifying early resistance and predicting successful treatment with CDK4/6i have yet to be investigated and elucidate an area of unmet clinical need.

Homeodomain transcription factors play a major role in regulating various cellular behavior, such as cell proliferation, cell differentiation, migration, and apoptosis.^5^ In our previous studies, we found that Barx2,^6^ Nkx6.1,^7^ and HOXC10^8^ homeodomain transcription factors can influence Hepatocellular carcinoma or GC progression. It was hypothesized that PAX proteins might inhibit terminal differentiation and apoptosis in issue‐specific stem cells, which results in the maintenance of these cells.^9^ Interestingly, this effect may be involved in the development and progression of cancer cells. Paired box 6 (PAX6) has been identified as a *Pax* gene, which encodes a homeobox and paired domain‐containing protein that binds DNA and acts as a regulator of transcription.^10^ Interestingly, PAX6 has been implicated as a cell cycle regulator by arresting cells in the G0/G1‐phase in human gliomas.^11^ PAX6 could exert regional control of cortical progenitor proliferation through direct repression of Cdk6 and hypophosphorylation of pRb.^12^ However, PAX6 can act as an oncogene for cancer stemness induction in lung adenocarcinoma.^13^ Therefore, PAX6 can act as a tumor suppressor or in an oncogenic manner, depending on the tissue type.^14^, ^15^, ^16^ Although PAX6 is upregulated in GC,^17^ its potential biological role and the underlying molecular mechanisms of PAX6 in GC have not yet been elucidated.

The Hippo signaling pathway has been implicated in various cancer‐related processes, such as tumorigenesis, cancer stem cell self‐renewal, and anti‐tumor drug resistance.^18^ Large tumor suppressor 1/2 (LATS1/2) is one of the major members of the Hippo pathway. Interestingly, activated LATS1/2 can phosphorylate yes‐associated protein (YAP) as well as the transcriptional coactivator with PDZ binding motif (TAZ), which participates in tumorigenesis.^19^ However, the effect of CDK4/6 inhibitor treatment is restricted by drug resistance; inactivating the Hippo pathway may provide an explanation.^20^ A recent study reported that loss of FAT1 inactivates the Hippo pathway and activates YAP/TAZ, which results in breast cancer cell resistance to CDK4/6i.^21^ There is a hypothesis that other related factors in the Hippo pathway could result in CDK4/6 inhibitor resistance; therefore, there is a need for further investigation of these possibilities. Hence, we investigated whether PAX6 alters the hypermethylated promoter of *LATS2* and suppresses the Hippo pathway to promote GC cell resistance to CDK4/6i

## 2 MATERIALS AND METHODS

### 2.1 Immunohistochemistry and immunofluorescence

A total of 218 primary GC samples with intact data were collected from the Sun Yat‐sen University Cancer Center (Guangzhou, China) between September 2002 and December 2004. The mean age of the patients was 62 years (range: 46–75 years). All patients were classified according to the American Joint Committee on Cancer and tumor node metastasis classification system. TMA (tissue microarray) was constructed and IHC (Immunohistochemistry) for sample staining was performed as described previously.^22^

We collected another eight, paired, fresh GC/adjacent noncancerous tissues, and PAX6 expression was detected by qRT‐PCR and western blot analysis. The enrolled patients were not subjected to any adjuvant therapy at the time of entry into the study. The follow‐up time was described previously.^22^

The immunoreactivity score of PAX6 was calculated by summation of intensity and staining scores.^22^ In this study, we obtained approval from the Institute Research Medical Ethics Committee of Sun Yat‐sen University.

### 2.2 Cell culture and reagents

Human gastric mucosal epithelial cell lines GES‐1 and GC cell lines (AGS, MKN45, N87, SNU‐1, and HGC27) were obtained from the American Type Culture Collection (USA). RPMI 1640 medium containing 10% FBS (Invitrogen, Thermo Fisher Scientific, MA, USA) was used to maintain cell lines and incubated at 37°C in a humidified 5% CO_2_ atmosphere. A list of the antibodies used is shown in Table S4.

### 2.3 Western blotting analysis

Indicated cells were incubated in CELLSTAR six‐well plates (Greiner Bio‐One, Frickenhausen, Germany), washed in PBS, and western blot analysis was conducted as previously described.^22^

### 2.4 Nuclear and cytoplasmic protein extraction

A protein extraction kit (Beyotime, China) was used to extract the proteins. Nuclear and cytoplasmic proteins were extracted using the Nuclear and Cytoplasmic Protein Extraction Kit (Beyotime, China), according to the manufacturer's instructions.

### 2.5 qRT‐PCR

Total RNA was extracted from cells or tissues using TRIzol reagent (Thermo Fisher Scientific). The procedure was conducted as described previously.^22^

### 2.6 Construction of stable overexpression and knockdown GC cell lines

PAX6 lentiviral activation particles (PAX6 transcript variant 1), PAX6 shRNA pGFP‐C‐shLenti vector, CCND1, and LATS2 lentiviral activation particles or or shRNA pGFP‐C‐shLenti vectors, and scrambled particles were purchased from GeneChem (Shanghai, China). Transfection was performed as previous described.^22^ The sequences are listed in Table S5.

### 2.7 Flow cytometry assay

For apoptosis analysis, cells were collected and detected using the Annexin V‐Fluos Staining Kit (Roche, Basel, Switzerland) according to the manufacturer's protocol. Flow cytometry was performed using FACScan (Becton‐Dickinson, Bedford, MA, USA).

### 2.8 Mitochondrial membrane potential assay

MitoPT JC‐1 Assay Kit (ImmunoChemistry Technologies, Bloomington, USA) was used to examine mitochondrial membrane potential, and the procedure was conducted according to the manufacturer's instructions.

### 2.9 MTS assay

Cell proliferation and viability were determined using the MTS assay. The indicated cells were embedded in CELLSTAR 96‐well plates (Greiner Bio‐One, Frickenhausen, Germany). Forty‐eight hours after incubation, the cells were mixed with MTS reagent. The measurements were conducted according to the manufacturer's instructions.

### 2.10 Foci formation assay

A total of 1 × 10^3^ GC cells were planted in six‐well plates. After culturing for 2 weeks, the cell colonies were counted using crystal violet staining.

### 2.11 Cell cycle distribution analysis

|A total of 1 × 10^6^ indicated cells were resuspended in 200 ml of citrate buffer (0.25 M sucrose, 40 mM sodium citrate; pH 7.6). The procedure was conducted as previously described.^4^

### 2.12 Viability assay

The CellTiter‐Blue Cell Viability Assay was obtained from Promega Corporation (Madison, WI, USA), the procedure was conducted as previously decribed.^21^

### 2.13 CHX‐chase assay

Cells (1 × 10^5^) were seeded and transfected with 0.5 μg of pCH5003 and 3 μl of polyethyleneimine (PEI) (1 mg/ml stock solution in water; pH 7.0). After 48 h, the cells were treated with CHX (final concentration: 100 μg/μl), and lysed in RIPA buffer (89900, Thermo Fisher Scientific) at the indicated times. Cell lysates were then incubated and centrifuged. The supernatants were obtained, and protein concentrations were measured using Bradford assays. Finally, DNMT1 or GAPDH was examined by immunoblotting.

### 2.14 Co‐immunoprecipitation assay

Anti‐PAX6 and anti‐DNMT1 were employed to co‐precipitate interacting proteins in indicated cell lysates. The target protein was drawn down with electrophoresis bands and detected by western blotting; the procedure was conducted according to the manufacturer's protocol.

### 2.15 Chromatin immunoprecipitation assay

A ChIP assay was performed using the EZ‐Magna ChIP kit (Millipore Corp, MA, USA) according to the manufacturer's instructions. In brief, chromatin was extracted, and DNA was cut into 0.2–1 kb fragments. Indicated cells were cross‐linked using 1% formaldehyde, and chromatin was immunoprecipitated with the indicated antibody. The ChIP DNA thus obtained was analyzed using RT‐qPCR.

### 2.16 Terminal deoxynucleotidyl transferase‐mediated deoxyuridine triphosphate nick end labeling assay

AGS‐PAX6‐vector and AGS‐PAX6‐LATS2 were seeded into a Nunc Lab‐Tek chamber slide system (Thermo Fisher Scientific). After incubation for 24 h, the cells were treated with palbociclib for 48 h. The triphosphate nick end labeling (TUNEL) assay (Roche Diagnostics, Mannheim, Germany) was used to identify apoptotic cells, as previously described.^23^ Fluorescence was examined using a Leica DMI6000 B microscope (Leica, Wetzlar, Germany).

### 2.17 Microarray and gene expression analysis

Gene expression data were analyzed using log_2_ transformation. Significant differences between the expression levels were analyzed by Student's *t*‐test. The procedure was conducted as described previously described.^22^

### 2.18 DNMT activity

Nuclei were isolated from the AGS‐vector, AGS‐PAX6, HGC27‐shcontrol, and HGC27‐shPAX6 cells using an EpiQuik Nuclear Extraction Kit I (Epigentek Group Inc. USA). DNMT activity of the nuclear fractions (5 μg protein per sample) was examined by an EpiQuik DNMT Activity/Inhibition Assay Ultra Kit (Epigentek Group Inc). Pierce BCA Protein Assay Kit (Thermo Fisher Scientific) was employed to detect protein concentrations.

### 2.19 TCGA

Clinical data and normalized gene expression data of TCGA samples were derived from the TCGA dataset (https://portal.gdc.cancer.gov/).

### 2.20 Animal experiments

The mouse studies were approved by the Ethics Committee of the First Affiliated Hospital of Sun Yat‐sen University, and the mice were purchased from the Experimental Animal Center of Sun Yat‐sen University. Indicated cells were inoculated subcutaneously under anesthesia (1 × 10^6^ cells) in 35‐day‐old male nude mice. Briefly, HGC27‐shcontrol, HGC27‐shPAX6, AGS‐vector, and AGS‐PAX6 were injected, and the tumor sizes of all mice were monitored every other day. When raised for 35 days, the indicated mice group was treated daily with 150 mg/kg palbociclib, 5‐Aza 5 mg/kg, or both for 16 consecutive days via oral gavage. The mice were euthanized under anesthesia, and the tumors were isolated and measured. Tumor volume was calculated as: tumor volume (mm^3^) = 0.5 × l × w^2^, where l refers to the length (long axis) and w refers to the width (short axis) of the tumor.

### 2.21 Statistical analysis

SPSS v17 software was employed to perform statistical analyses. The data in this study are expressed as the mean ± standard error of the mean from at least three independent experiments. Student's *t*‐test was used to compare quantitative data between groups. The c2 or Fisher's exact test was used to analyze categorical data. Correlations between protein expression levels were analyzed using Spearman's rank analysis. Differences in OS and DFS were analyzed using Kaplan‐Meier and log‐rank tests. A Cox proportional hazards model was used to determine the regression analyses of survival and recurrence. A linear regression test was used to analyze correlations between gene expression levels. Statistical significance was set at *p *< 0.05.

## 3 RESULTS

### 3.1 Overexpressed PAX6 is associated with poor outcomes in GC

**FIGURE 1:**
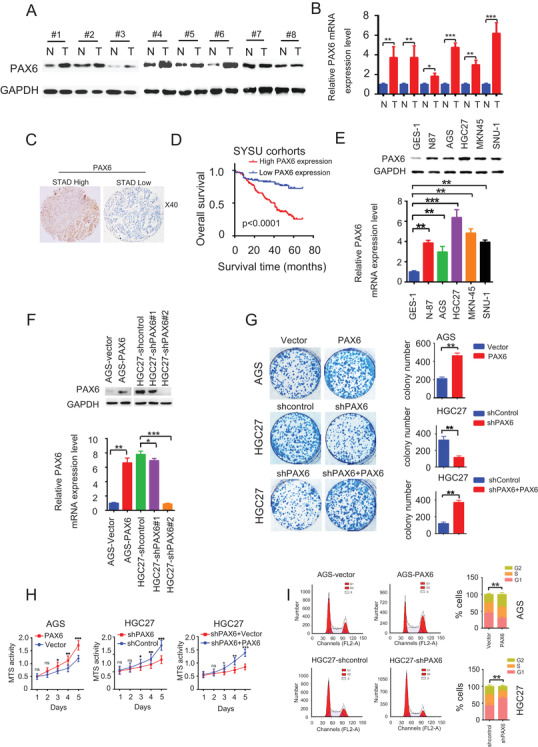
PAX6 is associated with GC prognosis and can promote GC cell progression. (A) Representative western blots of PAX6 protein expression in paired GC tissues and adjacent non‐tumor tissues. Three independently repeated experiments were performed with similar results. (B) Representative qRT‐PCR of PAX6 mRNA expression in paired GC tissues and adjacent non‐tumor tissues. Three independently repeated experiments were performed with similar results. Student's *t*‐test: *p < 0.05. **p < 0.01, ***p < 0.001. (C) Representative IHC images of PAX6 protein expression in paired GC tissues and adjacent non‐tumor tissues from TMA; magnification 40X. (D) OS association with the expression levels of PAX6 in the 218 patients in the GC patient. (E) Representative western blots of PAX6 protein expression (upper panel) or qRT‐PCR of PAX6 mRNA expression (lower panel) in human GC cell lines, and human normal gastric epithelial cells GES‐1. Three independently repeated experiments were performed with similar results. Student's *t*‐test: *p < 0.05. **p < 0.01, ***p < 0.001. (F) Representative western blots of PAX6 protein expression (upper panel) or qRT‐PCR of PAX6 mRNA expression (lower panel) in GC cells transduced with PAX6 or shPAX6, and their paired control vectors. Three independently repeated experiments were performed with similar results. Student's *t*‐test: *p < 0.05. **p < 0.01, ***p < 0.001. (G) Representative photographs of plate wells and microscopy images of AGS and HGC27 cells stained with crystal violet (left panel). The number of colonies is presented as a graph (right panel). Scale bar, 100 μm. Student's *t‐*test: *p < 0.05. **p < 0.01, ***p < 0.001. (H) MTS assays of indicated GC cells. Three independently repeated experiments were performed with similar results. Student's *t*‐test: *p < 0.05. **p < 0.01, ***p < 0.001. (I) Cell cycle distribution of indicated cells. Three independently repeated experiments were performed with similar results. Chi‐square test: *p < 0.05, **p < 0.01, ***p < 0.001 Abbreviations: N, non‐tumor tissues; ns, not significant; T, tumor tissues.

Although it was reported that the expression of PAX6 is upregulated by hsa‐miR‐375 in GC,^17^ the biological function of PAX6 in GC remains unclear. To validate the expression levels of PAX6 in GC, qRT‐PCR and western blotting were used to analyze mRNA or protein expression levels of PAX6 in eight paired fresh GC tissues. PAX6 mRNA or protein was upregulated in six paired fresh GC samples compared to that in adjacent non‐tumor tissues (6/8, 75%) (Figures 1A and 1B). To understand the role of PAX6 in GC prognosis, IHC was used to analyze PAX6 expression levels in 218 GC patients with intact clinical follow‐up data. In adjacent noncancerous tissues, the staining of PAX6 was weak or absent. However, we found that the expression levels of PAX6 were noticeably higher in GC tissues than in adjacent noncancerous tissues (Figure 1C). Overexpression of PAX6 was observed in 65.60% (143/218) of patients with GC (Table S1). Moreover, the expression levels of PAX6 were significantly correlated with multiple pathological variables (Table S1), including tumor size (*p* = 0.008), differentiation state (*p* = 0.003), clinical stage (*p* < 0.001), and metastasis (*p* = 0.001). Meanwhile, PAX6 expression was not found to be associated with age (*p* = 0.759) or sex (*p* = 0.289). Kaplan–Meier survival analyses revealed that the cumulative 5‐year overall survival (OS) rate was 72% in patients with low levels of PAX6 expression and 29% in patients with high levels of PAX6 expression (Figure 1D). Additionally, a multiple Cox regression analysis was performed (Table S2). The results revealed that PAX6 high expression levels were correlated with adverse OS (hazard ratio, 4.160; 95% CI, 2.364–7.322; *p* < 0.001). Tumor differentiation, tumor size, clinical stage, metastasis, and invasion depth were also associated with poor survival in patients with GC. In addition, the results of the multivariate analyses revealed that PAX6 expression level independently predicted poor OS (hazard ratio: 2.542; 95% CI: 1.411–4.579, *p* = 0.002), as well as the clinical stage, invasion depth, tumor differentiation, and metastasis (Table S3).

### 3.2 PAX6 promotes cell proliferation and cell cycle progression in GC cells

Next, the expression of PAX6 in GC cells was examined. Interestingly, we revealed that PAX6 levels were higher in GC cells than in human gastric epithelial cells GES‐1 (Figure 1E). We also found that PAX6 expression levels in AGS cells were lower than those in N87, MKN45, SNU‐1, and HGC27 cells, suggesting an expression variability of PAX6 in GC cell lines (Figure 1E). To establish whether and how PAX6 affected GC cells, gain‐ and loss‐of‐function studies were performed (Figure 1F). The PAX6 overexpression model was generated by ectopic expression of the PAX6 plasmid in AGS cells. The PAX6 knockdown model was generated by expressing PAX6 shRNA‐targeting plasmids in HGC27 cells. Notably, overexpressed PAX6 induced an increase in colony numbers compared to that in AGS‐vector cells, while knockdown of PAX6 achieved the opposite effect (Figure 1 G). Furthermore, MTS analysis demonstrated that overexpression of PAX6 increased MTS activity when compared with that of vector cells, and PAX6 knockdown contributed to decreasing MTS activity compared to that in sh‐control cells (Figure 1H). Taken together, these results reveal the role of PAX6 in promoting cell proliferation in GC cells.

Dysregulation of the cell‐cycle machinery contributes to uncontrolled cell proliferation.^24^ Therefore, we investigated whether PAX6 was involved in the regulation of cell cycle progression. It was noted that PAX6 overexpression reduced G1 cell cycle arrest, but PAX6 knockdown induced strong G1 cell cycle arrest (Figure 1I). This suggests that PAX6 can inhibit G1 cell cycle arrest in GC cells.

### 3.3 PAX6 promotes GC cell resistance to the CDK4/6 inhibitor

**FIGURE 2:**
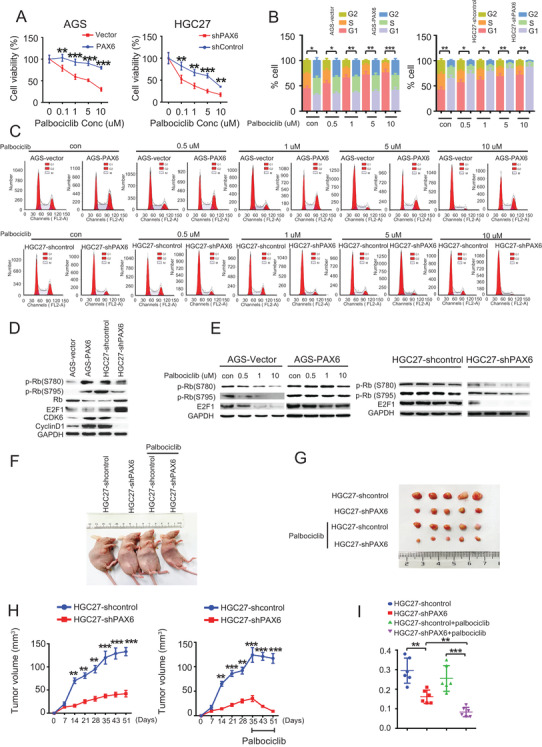
PAX6 drives palbociclib resistance in GC. (A) Viability of AGS‐PAX6 and HGC27‐shPAX6 versus control cells following treatment with indicated concentrations of palbociclib. Chi‐square test: *p < 0.05. **p < 0.01, ***p < 0.001. The palbociclib treatment duration was 8 days. (B and C) Cell cycle distribution of AGS‐PAX6, HGC27‐shPAX6, and their control cells after 72 h treatment with the indicated palbociclib doses. Mean ± standard deviation (SD); *n* = 3. Chi‐square test: *p < 0.05. **p < 0.01, ***p < 0.001; compared with no palbociclib (CV). (D) Representative western blot analysis of p‐Rb (S780), p‐Rb (S795), Rb, E2F1, CDK6, cyclin D1 in AGS‐PAX6 or HGC27‐shPAX6, and their control cells. GAPDH was used as the internal control. Three independently repeated experiments were performed with similar results. (E) Representative western blot analysis of p‐Rb (S780), p‐Rb (S795), and E2F1 in AGS‐PAX6 or HGC27‐shPAX6, and their control cells after 72 h treatment with the indicated palbociclib doses. GAPDH was used as the internal control. Three independently repeated experiments were performed with similar results. (F and G) Representative images of subcutaneous tumors derived from the indicated GC cells after treatment with palbociclib. (H) Tumor growth in mice subcutaneously injected with the indicated stable cell lines at the indicated time points. *n* = 5 mice per group. Palbociclib was treated on day 35, and tumor volume was monitored for 16 d. (I) Tumors derived from the indicated GC cells were weighed. *n* = 5 for each group. Student's *t*‐test: *p < 0.05. **p < 0.01, ***p < 0.001 Abbreviation: ns, not significant.

Actions of CDKs result in the transition of the cell cycle stage, and they are activated by interaction with their partner cyclins.^25^ Furthermore, of the three CDK4/6 inhibitors (palbociclib, ribociclib, and abemaciclib), palbociclib is one of the most widely used in clinics.^26^ Therefore, we investigated whether PAX6 affects CDK4/6 inhibitor efficacy in GC. Notably, AGS cells stably transduced with a PAX6 expression vector were relatively resistant to palbociclib (Figure 2A), abemaciclib (Figure S1a), and ribociclib (Figure S1a), and this effect was reversed when the shPAX6 vector was transduced into HGC27 cells (Figures 2A and S1a). However, upon exposure to increasing concentrations of palbociclib (0, 0.1, 1, 5, and 10 μM), the cell cycle of the AGS‐vector was completely inhibited, whereas that of AGS‐PAX6 cells was not, although palbociclib at 5–10 μM is not specific to CDK4 or CDK6.^27^ In line with this, the cell cycle of HGC27‐shPAX6 cells was promoted in contrast to that of the HGC27‐shcontrol cells (Figures 2B and 2C).

To examine whether drug resistance is due to insensitive CDK4/6 kinase activity, the expression of the CDK4/6 substrate was assessed after 24 h of drug exposure. Notably, ectopic PAX6 expression in AGS cells led to upregulated RB phosphorylation (S780 and S795), and cyclin D1, CDK6, and E2F1 were associated with a proportional decrease in RB, while PAX6 knockdown achieved the opposite effect (Figure 2D). These results indicate that PAX6 induced an increase in cyclin D1, which was previously reported to facilitate CDK4/6i resistance.^28^, ^29^ Furthermore, with palbociclib treatment, RB phosphorylation (S780 and S795), and E2F1 were completely blocked by drugs in the AGS‐vector cells; there was only partial inhibition of RB phosphorylation in AGS‐PAX6 cells. Consistent with this, upon exposure to palbociclib, RB phosphorylation (S780 and S795) and E2F1 were suppressed in HGC27‐shPAX6 cells but not in HGC27‐shcontrol cells (Figure 2E). Taken together, these data suggest that overexpressed PAX6 can confer resistance to palbociclib in GC cells *in vitro*.

Furthermore, the *in vivo* efficacy of palbociclib in human gastric HGC27‐shcontrol and HGC27‐shPAX6 xenograft models was studied. Palbociclib was administered orally (p.o.) daily for 4 weeks.^4^ First, the tumor volumes and weights in the HGC27‐shPAX6 without palbociclib group were lower than those in the HGC27‐shcontrol group without palbociclib (Figures 2F and 2G). The administration of palbociclib resulted in a delay in tumor growth, which persisted after treatment in the HGC27‐shPAX6 xenograft model (Figures 2h and 2I). Tumor volumes and weights of the HGC27‐shPAX6 mice administered palbociclib were significantly smaller than those of HGC27‐shcontrol treated with palbociclib, and tumor volumes and weights of the HGC27‐shPAX6 mice administered palbociclib were significantly lower than those of HGC27‐shPAX6 mice without palbociclib administration (Figures 2h and 2I). There were no clinical signs of toxicity in the palbociclib‐treated mice. These data support the premise that PAX6 deficiency drives the vulnerability of GC cells to the CDK4/6 inhibitor, palbociclib, *in vivo*.

### 3.4 PAX6 causes CDK4/6 inhibitor palbociclib resistance in a Cyclin D1‐dependent manner

**FIGURE 3:**
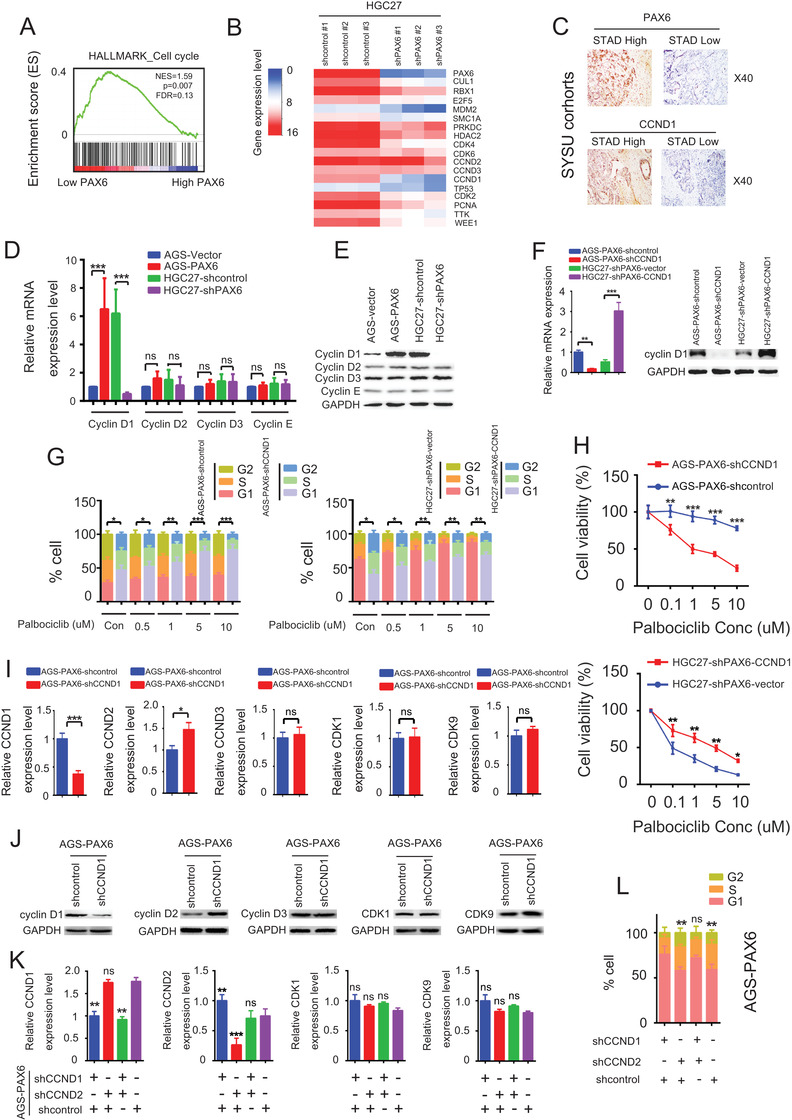
Cyclin D1 mediates PAX6, facilitating palbociclib resistance in GC. (A) GSEA plot of the association between gene sets positively correlated with PAX6 in the TCGA and STAD databases and the cell cycle signatures from the “Hallmark” gene set collection. (B) Relative mRNA levels of cell cycle‐related genes in HGC27‐shPAX6, and HGC27‐shcontrol cells detected by mRNA microarray. (C) Representative IHC images of PAX6 and CCND1 in 218 paired SYSU GC cohorts. Magnification 40X. (D) Relative mRNA expression level of cyclin D1, cyclin D2, cyclin D3, and cyclin E in AGS‐PAX6 and HGC27‐shPAX6, and their control cells. Student's *t*‐test: *p < 0.05, **p < 0.01, ***p < 0.001. (E) Western blot analysis of protein expression of cyclin D1, cyclin D2, cyclin D3, and cyclin E in AGS‐PAX6, HGC27‐shPAX6, and their control cells. (F) qRT‐PCR (left panels) and western blot analysis (right panels) of indicated cells. (G) Cell cycle distribution of AGS‐PAX6‐shCCND1, and HGC27‐shPAX6‐CCND1 after 72 h treatment with the indicated palbociclib doses. Mean ± standard deviation (SD); *n* = 3. Chi‐square test: *p < 0.05, **p < 0.01, ***p < 0.001; compared with AGS‐PAX6‐shcontrol or HGC27‐shPAX6‐vector. (H) Cell viability of AGS‐PAX6‐shCCND1, and HGC27‐shPAX6‐CCND1 after 72 h treatment with the indicated palbociclib doses. Mean ± standard deviation (SD); *n* = 3. Chi‐square test: *p < 0.05, **p < 0.01, ***p < 0.001; compared with AGS‐PAX6‐shcontrol or HGC27‐shPAX6‐vector. (I) Impact of CCND1 knockdown on indicated mRNA levels in AGS‐PAX6 cells. Transcript levels were determined by qPCR. Mean ± SD; *n* = 3. Student's *t*‐test: *p < 0.05. **p < 0.01, ***p < 0.001; compared with AGS‐PAX6‐shcontrol. (J) Impact of CCND1 knockdown on indicated protein levels in AGS‐PAX6 cells. Protein levels were determined by western blot analysis. Three independently repeated experiments were performed with similar results. (K) Effects of single and combined shRNA treatments on CCND2, and CCND1 RNA levels in AGS‐PAX6 cells. Transcript levels were analyzed in triplicate 48 h after electroporation by qPCR and were normalized to GAPDH. Mean ± standard deviation (SD), *n* = 3; Student's *t*‐test: *p < 0.05. **p < 0.01, ***p < 0.001; compared with shcontrol. (L) Cell cycle distribution of AGS‐PAX6 cells 48 h after electroporation with the indicated shRNA combinations. Chi‐square test: *p < 0.05, **p < 0.01, ***p < 0.001 Abbreviation: ns, not significant.

To address the underlying molecular mechanisms of GC cell susceptibility to the inhibition of CDK4/6 kinase activities, the key regulators of G1‐ to S‐phase cell cycle progression were analyzed. Interestingly, gene set enrichment analysis of the TCGA and STAD databases showed that PAX6 was positively correlated with the cell cycle signature (Figure 3A). Moreover, we noted that the top‐ranked genes affected in these cells upon PAX6 knockdown were cell cycle process‐related and closely mirrored each other in our microarray data (Figure 3B). We found that 101 genes were upregulated or downregulated after PAX6 knockdown, including 61 upregulated and 40 downregulated genes. Moreover, it was also found that CCND1 was positively correlated with PAX6, while CCND2 and CCND3 were not (Figure 3B). Therefore, whether PAX6 had an effect on CCND1 expression in GC cells was investigated. The relationship between PAX6 and CCND1 was analyzed by IHC (Figure 3C). Interestingly, PAX6 expression was positively correlated with CCND1 (*p* < 0.05). Next, CCND1, CCND2, CCND3, and CCNE1 expression levels in the cell line panel were examined. Notably, cyclin D1 was the only one differentially expressed in either AGS‐PAX6 or HGC27‐shPAX6 cells, compared to that in their parental cells, but not CCND2, CCND3, or CCNE (Figures 3d and 3E). Therefore, these observations suggest that overexpression of PAX6 upregulated CCND1 in GC cells.

Previous studies have demonstrated that cyclin D1 is involved in the early development of GC.^30^, ^31^ Cyclin D1 plays a role in the response to mitogenic signaling, which can promote progression through the G1‐S checkpoint of the cell cycle.^32^ As cyclin D1 can mediate the cell cycle and interact with CDK4/6, it is hypothesized that cyclin D1 can predict the response to CDK4/6 inhibition.^33^ Therefore, we hypothesized that cyclin D1 may mediate PAX6‐induced GC cell resistance to palbociclib. To test this, we first knocked down CCND1 in AGS‐PAX6 cells and overexpressed CCND1 in HGC27‐shPAX6 cells, as well as their parental cells (Figure 3F), and cell cycle analysis was performed to analyze whether CCND1 deficiency had an effect on G1‐ to S‐phase cell cycles with exposure to palbociclib. As expected, under exposure to palbociclib, CCND1 knockdown could significantly increase the percentage of cells in G0/G1 phase compared to that in AGS‐PAX6‐shcontrol cells (which was once decreased by overexpressed PAX6) while CCND1 overexpression resulted in the opposite (Figure 3 G) by supporting cyclin D1 mediated PAX6‐induced cell cycle progression. Furthermore, we confirmed that CCND1 was required for GC cell viability following exposure to palbociclib in AGS‐shPAX6 cells (Figure 3H).

Since a single D cyclin deficiency can be compensated by the other D cyclins,^34^ we decided to examine the transcript levels of CCND2, CCND3, CDK1, and CDK9 after CCND1 knockdown. Of note, transcript levels of CCND2 increased more than 1.5‐fold while other genes did not (Figures 3i and 3J). However, simultaneous knockdown of CCND1 and CCND2 did not enhance the effects of CCND1 knockdown alone (Figures 3k and 3L). Taken together, the loss of CCND1 could not be functionally compensated for by the altered expression of other G1 CDK‐CCND complexes.

### 3.5 PAX6 suppressed the Hippo pathway

**FIGURE 4:**
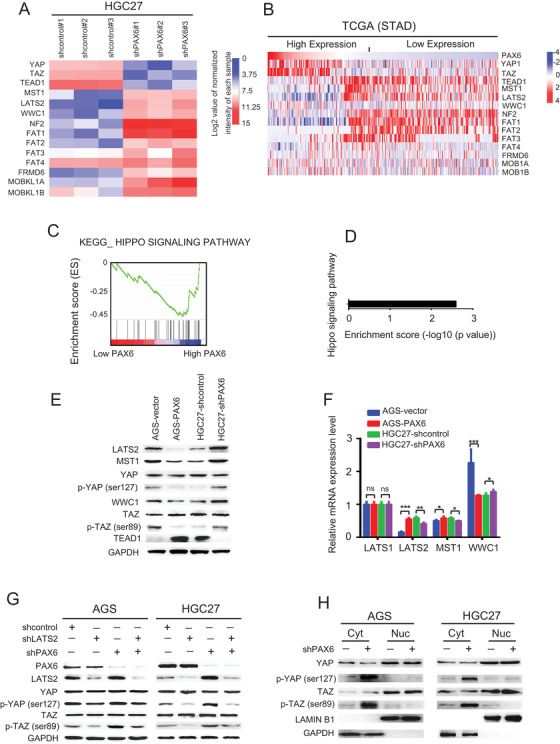
Hippo signaling pathway is regulated by PAX6 in GC. (A) Relative mRNA levels of Hippo signaling pathway‐related genes in HGC27‐shPAX6 and HGC27‐shcontrol cells detected by gene microarray. (B) Relative mRNA levels of Hippo signaling pathway‐related genes in the TCGA and STAD databases. (C) GSEA plot of the association between gene sets positively correlated with PAX6 in the TCGA and STAD databases, and the Hippo signaling pathway signatures from “Hallmark” gene set collection. (D) Kyoto Encyclopedia of Genes and Genomes (KEGG) analysis of PAX6‐regulated signaling pathways in gene microarray. (E) Western blot analysis of Hippo signaling pathway‐related protein expression in indicated cells. Three independently repeated experiments were performed with similar results. (F) qRT‐PCR analysis of Hippo signaling pathway‐related mRNA expression in indicated cells. Three independently repeated experiments were performed with similar results. Student's *t*‐test: *p < 0.05, **p < 0.01, ***p < 0.001. (G) Representative western blots showing the levels of PAX6, LATS2, YAP, and p‐YAP (ser127) in AGS and HGC27 transfected with shcontrol, shLATS2, or shPAX6. GAPDH was used as the internal control. The results show one representative of three similar experiments. (H) Immunoblot analysis of YAP, and p‐YAP (ser127) in the cytoplasmic and nuclear fractions of AGS, and HGC27 cells. LAMINB1 was used as the internal control Abbreviation: ns, not significant.

To explore the mechanisms by which PAX6 regulates CCND1 expression, mRNA microarray analysis was performed to examine the potential targeted pathway of PAX6. Notably, it was found that the expression levels of Hippo pathway members were activated in PAX6 knockdown GC cells when compared to sh‐control GC cells (*p* = 0.0025) (Figures 4A and 4D). This result was further confirmed in TCGA (Figures 4B and 4C), which suggests that PAX6 is associated with the Hippo signaling pathway. Furthermore, multiple canonical transcriptional targets of the Hippo signaling pathway were assessed by qPCR and western blotting in parental and PAX6 overexpressed or knocked down cells. While the expression of the Hippo signaling pathway target, LATS1, was unaffected by PAX6 loss (Figure 4F), the marked upregulation of LATS2, MST1, p‐YAP, and WWC1 was detected, consistent with the activation of Hippo signaling. Consistently, a marked downregulation of LATS2, MST1, p‐YAP, and WWC1 was observed (Figures 4E and 4F). Taken together, these findings indicate that PAX6 profoundly inhibits the Hippo signaling pathway in GC cells.

LATS1/2 kinase activation or YAP/TAZ inactivation represents the major functional output of the Hippo pathway.^35^ When LATS1/2 kinases are activated, they can directly phosphorylate YAP and TAZ, which mediate the transcriptional output of the Hippo pathway.^36^ We next investigated whether LATS2 is essential for the PAX6 regulated Hippo signaling pathway. Notably, we found that simultaneous knockdown of PAX6 and LATS2 prominently reduced the phosphorylation of YAP promoted by the loss of PAX6 alone in both AGS and HGC27 cells (Figure 4 G). However, the response of p‐YAP to the abrogation of LATS2 in AGS cells was more modest than that in HGC27 cells. In light of the different expression levels of PAX6 in AGS and HGC27 cells, this result suggests that the effect of LATS2 on the downstream effectors of the Hippo signaling pathway is PAX6‐dependent. Furthermore, strong nuclear YAP expression was evident at baseline, while PAX6 knockdown resulted in a decrease in nuclear YAP and a dramatic accumulation of phosphorylated YAP‐S127 in the cytosol (Figure 4H). YAP is transcriptionally activated when translocated into the nucleus.^37^, ^38^ YAP dephosphorylation promotes YAP nuclear translocation, which results in YAP/TEAD transcriptional activation.^39^ Taken together, these data suggest that PAX6 can inactivate the Hippo signaling pathway.

### 3.6 PAX6 facilitated CDK4/6 inhibitor resistance by suppressing the Hippo pathway

**FIGURE 5:**
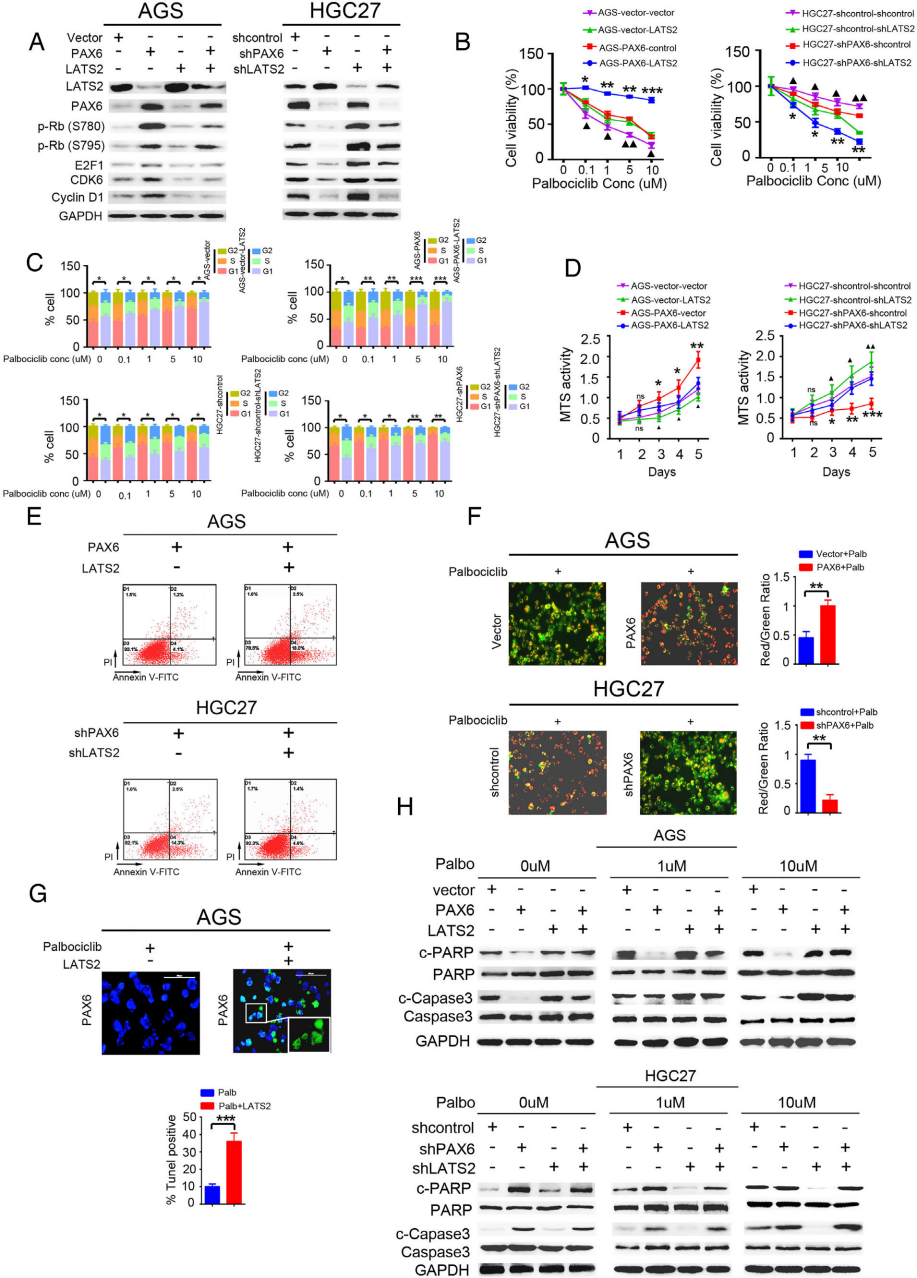
Inhibition of the Hippo signaling pathway facilitates palbociclib resistance in GC. (A) Western blot analysis of LATS2, PAX6, p‐Rb (S780), p‐Rb (S795), E2F1, CDK6, and cyclin D1 in indicated cells. Three independently repeated experiments were performed with similar results. (B) Cell viability of indicated cells following treatment with indicated concentrations of palbociclib. Chi‐square test: *p < 0.05, **p < 0.01, ***p < 0.001. (C) Cell cycle distribution of indicated cells after 72 h treatment with the indicated palbociclib doses. Mean ± standard deviation (SD); *n* = 3; Chi‐square test: *p < 0.05, **p < 0.01, ***p < 0.001; compared with no palbociclib (CV). (D) MTS assays of indicated GC cells. Three independently repeated experiments were performed with similar results. Student's *t*‐test: *p < 0.05. **p < 0.01, ***p < 0.001. (E) Representative flow cytometry dot plots of apoptosis after Annexin V‐FITC/PI dual staining in indicated cells. Three independently repeated experiments were performed with similar results. (F) JC‐1 staining. The red:green fluorescence ratio reflects changes in the mitochondrial membrane potential of GC cells in the AGS‐vector, AGS‐PAX6, HGC27‐shcontrol, and HGC27‐shPAX6 with treatment of palbociclib (10 μM). Student's *t*‐test: *p < 0.05. **p < 0.01, ***p < 0.001. (G) TUNEL staining of AGS‐PAX6 cells with LATS2 overexpressed or not with treatment with palbociclib (10 μM). Nuclei were stained with DAPI. The number of TUNEL‐positive cells was significantly greater in the AGS‐PAX6‐LATS2 group than in the AGS‐PAX6‐vector group; Chi‐square test: *p < 0.05, **p < 0.01, ***p < 0.001. (H) Representative western blot analysis of apoptosis‐related markers in indicated cells with indicated palbociclib doses. Three independently repeated experiments were performed with similar results Abbreviation: ns, not significant.

Previous studies have indicated that YAP promotes cell proliferation, and exogenous LATS2 inhibits cell proliferation by inducing YAP phosphorylation.^40^ Furthermore, cell cycle‐regulating genes, including CCND1 and FOXM1, are induced by YAP and, thus, play a role in cell cycle regulation.^41^ In addition, several studies have identified a set of Rb/E2F‐regulated genes that predict the response to CDK4/6i.^42^, ^43^ Therefore, we tested whether the Hippo pathway signaling‐mediated PAX6 promoted CDK4/6 inhibitor resistance. Notably, we showed that the expression levels of pRb (S780), pRb (S795), and E2F1 were downregulated in AGS‐PAX6‐LATS2 when compared with the AGS‐PAX6‐vector. In contrast, pRb (S780), pRb (S795), and E2F1 expression levels were upregulated in HGC27‐shPAX6‐shLATS2 when compared with HGC27‐shPAX6‐shcontrol (Figure 5A). Additionally, the downregulation of cyclin D1 was identified as a result of LATS2 overexpression. These data suggest that the dysregulation of LATS2, a critical component of the Hippo pathway, influences CDK4/6 kinase activity. More importantly, the treatment of AGS‐PAX6‐LATS2 cells with CDK4/6i palbociclib displayed reduced resistance when compared to that in AGS‐PAX6‐control cells, as demonstrated by lower cell viability (Figure 5B), higher G1 cell cycle arrest (Figure 5C), and lower MTS activity (Figure 5D). In contrast, treatment with shLATS2‐expressing shPAX6 cells achieved the opposite results (Figure 5B–D). These data indicate that LATS2 drives PAX6‐mediated palbociclib resistance in GC cells.

The major mechanism of action of CDK4/6i is to block the cell cycle at the G1 to the S transition phase, which could prevent the proliferation of cancer cells.^44^ Notably, treatment of acute myeloid leukemia cells with palbociclib showed an increased G0/G1 phase and increased apoptotic cells,^45^ suggesting that palbociclib can promote cell apoptosis. Furthermore, YAP promotes Skp2 cytoplasmic retention, which can over‐degrade the pro‐apoptotic factor, FoxO1/3, resulting in apoptosis being inhibited.^46^ This suggests that suppression of the Hippo signaling pathway can inhibit cell apoptosis. Therefore, to gain further insight into the underlying mechanism of CDK4/6i resistance in GC cells, we tested whether PAX6 affects CDK4/6i‐induced GC cell apoptosis. The Hippo signaling pathway mediating PAX6‐induced cell apoptosis was evaluated first. As expected, overexpression of LATS2 promoted apoptosis in AGS‐PAX6 cells and knocked down LATS2 in HGC27‐shPAX6 cells achieved the opposite (Figure 5E). A JC‐1 assay was performed to test whether PAX6 affects CDK4/6i‐induced cell apoptosis. Interestingly, it was found that CDK4/6i inducing AGS cell apoptosis was compromised by the overexpression of PAX6, whereas shPAX6‐expressing HGC27 cells showed an increased percentage of apoptotic cells in the presence of CDK4/6i. This further confirmed that PAX6 promotes CDK4/6i resistance in GC cells (Figure 5F). To further confirm whether the Hippo signaling pathway is the major effector of PAX6‐mediated inhibition of cell apoptosis in the presence of CDK4/6i, we performed a TUNEL assay. Cell apoptosis was also promoted when LATS2 was overexpressed in AGS‐PAX6 cells with palbociclib treatment when compared with AGS‐PAX6 cells without overexpressed LATS2 (Figure 5 G). Similarly, pro‐apoptotic factor, c‐PARP, and c‐Caspase3, levels were decreased by PAX6 overexpression in AGS cells with or without palbociclib treatment, which was compromised by LATS2 overexpression. In contrast, PAX6 knockdown increased c‐PARP and c‐Caspase3 levels with or without the presence of palbociclib (Figure 5H). However, PAX6 knockdown‐induced c‐PARP and c‐Caspase3 augmentation was abrogated in LATS2 knocked down cells (Figure 5H), indicating that PAX6 could inhibit CDK4/6i‐induced cell apoptosis by suppressing the Hippo signaling pathway. In summary, these data demonstrate that the PAX6/LATS2 axis drives palbociclib resistance, not only by promoting cell proliferation and the cell cycle, but also by inhibiting cell apoptosis.

### 3.7 PAX6 regulates the Hippo pathway via DNA methylation

**FIGURE 6:**
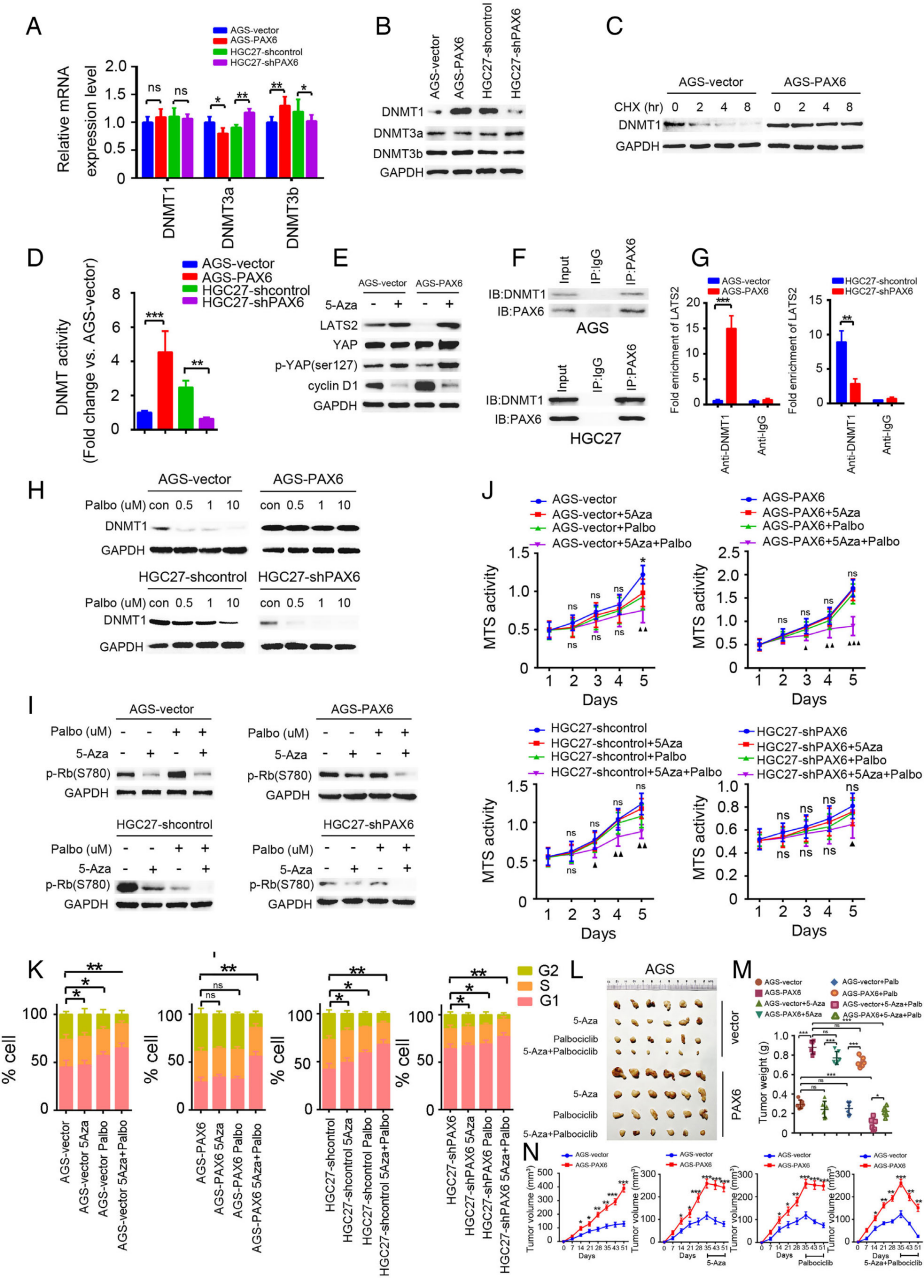
Inhibition of PAX6‐induced DNA methylation improves the response to palbociclib. (A) Representative qRT‐PCR of mRNA expression of DNMT1, DNMT3a, and DNMT3b in indicated cells. Three independently repeated experiments were performed with similar results. Student's *t*‐test: *p < 0.05. **p < 0.01, ***p < 0.001. (B) Representative western blot analysis of protein expression of DNMT1, DNMT3a, and DNMT3b in indicated cells. Three independently repeated experiments were performed with similar results. (C) CHX was used to measure the half‐life of DNMT1 protein in AGS‐vector cells or AGS‐PAX6 cells. Three independently repeated experiments were performed with similar results. (D) DNMT activity of indicated cells when compared with AGS‐vector cells. Three independently repeated experiments were performed with similar results. Student's *t*‐test: *p < 0.05. **p < 0.01, ***p < 0.001. (E) Representative western blot analysis of LATS2, YAP, p‐YAP(ser127), and cyclin D1 in AGS‐vector and AGS‐PAX6 cells with or without 5‐Aza. GAPDH was used as internal control. Three independently repeated experiments were performed with similar results. (F) Co‐IP assay of PAX6 and DNMT1 in indicated cells. Three independently repeated experiments were performed with similar results. (G) Effect of DNMT1 on the LATS2 promoter region determined by ChIP in indicated cells. Three independently repeated experiments were performed with similar results. (H) Representative western blot analysis of DNMT1 protein expression in indicated cells with indicated palbociclib concentrations. Three independently repeated experiments were performed with similar results. (I) Representative western blot analysis of p‐Rb (S780) expression in indicated cells with palbociclib, 5‐Aza, or a palbociclib/5‐Aza combination. Three independently repeated experiments were performed with similar results. (J) MTS assay of indicated cells with palbociclib, 5‐Aza, or a palbociclib/5‐Aza combination. Student's *t*‐test: *p < 0.05. **p < 0.01, ***p < 0.001. (K) Cell cycle distribution of indicated cells with palbociclib, 5‐Aza, or a palbociclib/5‐Aza combination. Chi‐square test: *p < 0.05, **p < 0.01, ***p < 0.001. (L) Tumor growth in mice subcutaneously injected with the indicated stable cell lines treated with 5‐Aza, palbociclib, or both. (M) Tumors derived from the indicated GC cells were weighed. *n* = 6 for each group. Student's *t*‐test: *p < 0.05. **p < 0.01, ***p < 0.001. (N) Tumor growth in mice subcutaneously injected with the indicated stable cell lines at the indicated time points. *n* = 6 mice per group. 5‐Aza, Palbociclib or palbociclib/5‐Aza combination was treated on day 35, and tumor volume was monitored for 16 d Abbreviation: ns, not significant.

The results of this study indicate that PAX6 regulates LATS2 expression. However, the underlying mechanism of PAX6 regulation by LATS2 remains unclear. Previous studies have mainly focused on the association between PAX6 promoter methylation and cancer prognosis.^47^ However, a recent study reported that many epigenetic factor genes are transcriptionally regulated by PAX6,^48^ indicating that PAX6 can regulate DNA methylation. Therefore, we investigated whether PAX6 can regulate LATS2 through DNA methylation. Notably, it was found that the amount of DNMT1 protein increased with PAX6 overexpression (Figure 6B) but not mRNA expression levels of DNMT1 (Figure 6A), suggesting that PAX6 can stabilize DNMT1 protein, which was further confirmed by CHX assays (Figure 6C). In AGS‐PAX6 cells, DNMT1 activity increased compared to that in AGS‐vector cells, whereas DNMT1 activity was decreased by PAX6 knockdown in HGC27 cells (Figure 6D). Furthermore, to examine whether PAX6 can regulate LATS2 via DNA methylation, 5‐Aza was employed, which is a potent inhibitor of DNA methylation.^49^ Notably, the LATS2 expression level was increased in AGS‐PAX6 cells treated with 5‐Aza compared to AGS‐PAX6 cells without 5‐Aza treatment (Figure 6E). Interestingly, AGS‐PAX6 cells were found to be more sensitive to 5‐Aza than the AGS‐vector cells (Figure 6E). Moreover, we found that PAX6 could bind to the LATS2 promoter but not to CCND1 through DNMT1 (Figures 6F, 6G, and S1b). These data demonstrate that PAX6 regulates LATS2 expression by stabilizing DNMT1 protein.

A therapeutic strategy was investigated to overcome PAX6‐induced resistance to CDK4/6i through demethylation. The results of this study showed that PAX6 promotes CDK4/6i resistance by suppressing LATS2, and DNMT1 activity provides a major link between PAX6 and LATS2. In this study, we investigated whether a DNA methylation inhibitor augments the CDK4/6i response, which is inhibited by PAX6. In addition, the effect of CDK4/6i on DNMT1 expression was examined. Notably, it was found that DNMT1 protein expression was prominently inhibited in the presence of an increasing concentration of palbociclib in control cells compared to PAX6 overexpressed cells. Furthermore, knockdown of PAX6 augmented the effect of CDK4/6i on DNMT1 protein expression levels (Figure 6H). These data suggest that CDK4/6i can inhibit DNA methylation, which is consistent with the results of previous studies.^50^, ^51^ This further confirmed that PAX6 can induce CDK4/6i resistance. To test whether a demethylation agent could improve PAX6‐induced CDK4/6i resistance, the response of CDK4/6i palbociclib and 5‐Aza in AGS GC cells transfected with vector or PAX6, and in HGC27 GC cells with or without PAX6 knockdown, was compared. Notably, treatment with 5‐Aza decreased pRb (S780) compared to cells without 5‐Aza treatment, in control cells, in HGC27‐shPAX6 cells, and in AGS‐PAX6 cells. Furthermore, it was found that combining 5‐Aza with CDK4/6i prominently decreased pRB (S780) in control cells and HGC27‐shPAX6 cells, but there was no marked reduction in AGS‐vector cells (Figure 6I). Similarly, a greater decrease in MTS activity (Figure 6J) and an increase in cell cycle arrest (Figure 6K) in cells cotreated with CDK4/6i and 5‐Aza was found. Interestingly, we found that DNMT1 could increase pRB (S780) in AGS‐PAX6 or HGC27‐shPAX6 cells treated with both 5‐Aza and CDK4/6i, suggesting that 5‐Aza might alleviate palbociclib resistance in a PAX6 dependent manner through DNMT1 inhibition. Notably, DNMT1 could reverse the effect of the combination of 5‐Aza and CDK4/6i in alleviating PAX6‐induced palbociclib resistance, which demonstrated that 5‐Aza might alleviate palbociclib resistance in a PAX6 dependent manner through DNMT1 inhibition (Figure S1c). Moreover, combining 5‐Aza with CDK4/6i resulted in greater inhibition of AGS‐PAX6 tumor growth in mice than the single 5‐Aza treated group or CDK4/6i treated group, and this effect was augmented in the AGS‐vector group (Figures 6L–6N). Taken together, these results suggest that 5‐Aza can overcome PAX6‐ induced CDK4/6i resistance.

## 4 DISCUSSION

CDK4/6i has remarkable clinical utility in the treatment of cancer.^52^ A total of 7% and 12% of GCs exhibit CCND1 or CCNE1 alterations in TCGA, respectively.^4^ It has also been reported that cyclin D and E are highly expressed in GC and are associated with clinical outcomes.^4^ Likewise, dysregulation of the cell cycle plays an important role in GC initiation, progression, and therapeutic response.^53^ Taken together, these results suggest that CDK4/6 is an attractive therapeutic target for GC. However, the intrinsic or acquired resistance to CDK4/6i in many malignancies is widespread and poorly understood, including in GC. The present study revealed the mechanisms of CDK4/6i resistance in GC associated with overexpressed PAX6, a transcriptional factor that can epigenetically inactivate the Hippo signaling pathway, and, therefore, activate cyclin D1 to facilitate CDK4/6i resistance. Furthermore, 5‐Aza, a potent inhibitor of methylation, can overcome PAX6‐induced CDK4/6i resistance in GC.

Our previous studies have reported the role of homeobox genes in cancer progression.^6^, ^7^, ^8^ PAX6, a homeobox gene, is an essential transcription factor involved in embryonic development.^10^ Several previous studies have reported conflicting functions of PAX6 in different carcinomas, suggesting a tissue context‐dependent role of PAX6.^10^, ^11^, ^30^ In this study, it was found that PAX6 was negatively associated with the survival of patients with GC, indicating an oncogenic role of PAX6 in GC, which is consistent with the previously reported role of PAX6 in lung adenocarcinoma and pancreatic cancer.^13^ Furthermore, these results were further confirmed by the finding that PAX6 promoted GC cell proliferation and the cell cycle *in vitro*. The role of PAX6 in the cell cycle is of particular interest. Interestingly, PAX6 could maintain astrocytic glioma cells in the G1 phase of the cell cycle,^54^ suggesting that PAX6 promotes cell cycle arrest in glioma. However, based on the tissue context‐dependent role of PAX6 and its relationship with the poor prognosis of patients with GC, we hypothesized that PAX6 could facilitate the cell cycle in GC cells. The results of the current study confirm this hypothesis. The oncogenic role of PAX6 in GC may prove valuable for early diagnosis, and implies that more potent inhibitors of PAX6 may be valuable as strategies for GC treatment.

The interaction of cyclins with their partner serine/threonine CDKs can regulate the cell cycle. The importance of CDKs in the cell cycle was first elucidated by the discovery of cdc28 and cdc2 in budding and fission yeast, respectively.^55^ Targeting the activity of CDKs as a strategy to treat cancer is a concept that was first proposed when it was established that cell division is driven by these kinases.^56^, ^57^ Our data show that PAX6 can promote the cell cycle. However, whether PAX6 is implicated in CDK4/6i resistance remains unknown. In the present study, we revealed that PAX6 could facilitate CDK4/6i resistance in GC cells. Therefore, we were interested in the downstream of PAX6 in GC. A previous study revealed that Notch1 suppresses phosphor (p‐p38) and activates PAX6 expression, ultimately promoting cell proliferation via cyclin D1. Further, cyclin D1 is capable of transcriptionally upregulating PAX6,^58^ suggesting that expression of PAX6 is related to cyclin D1. As cyclin D1 plays a role in cell cycle mediation and its interplay with CDK4/6, it is hypothesized that cyclin D1 may predict the response to CDK4/6 inhibition. Notably, previously reported CDK4/6i sensitizers, including endocrine therapy in ER+ breast cancer,^59^ and MDM2i in human melanomas^24^ limit D‐type cyclins expression.^60^ Therefore, the role of cyclin D1 in PAX6‐mediated GC cells in CDK4/6i resistance is of particular interest. CCND1, which encodes cyclin D1, was amplified in approximately 17.4% of GC.^61^ Cyclin D1 is frequently overexpressed in the early stages of gastric carcinomas.^62^ Our data suggest that PAX6 can promote CDK4/6i resistance via cyclin D1, further confirming that cyclin D1 could be a CDK4/6i resistance sensitizer. This suggests that suppression of either PAX6 or cyclin D1 is a viable approach for improving CDK4/6i responses.

Beyond TEAD factors, YAP/TAZ can also cooperate with other transcriptional partners, including SMADs,^63^ T‐box transcription factor 5 (TBX5),^64^ and RUNT‐related transcription factors (RUNX1 and RUNX2).^65^ In our study, PAX6 suppressed LATS2 and activated YAP, further confirming that the transcription factor PAX6 is negatively correlated with Hippo signaling. Notably, activated YAP/TAZ results in resistance to chemotherapeutics or molecular targeted agents, including MEK‐, BRAF‐, and HER2‐directed agents.^66^ YAP, a key component of the Hippo signaling pathway can directly induce the transcription of CCND1 in cooperation with the TEAD transcription factor.^67^ Furthermore, LATS2 inhibition resulted in dramatic upregulation of cyclin D1.^68^ Although our data showed that PAX6 could upregulate cyclin D1 by suppressing LATS2, these data raise the question as to whether LATS2 might mediate PAX6, inducing CDK4/6i resistance. A recent study indicated that suppressed Hippo signaling by FAT1 could promote CDK4/6i resistance,^21^ suggesting that Hippo signaling is involved in CDK4/6i resistance. Of note, it remains to be understood which Hippo signaling pathway‐specific effector connects to CDK4/6i resistance, and this may yield additional therapeutic considerations for ultimately drugging this pathway in patients. A recent study reported that MDM2i could render resistant melanomas responsive to CDK4/6i by stabilizing p53.^28^ In addition, LATS2 can stabilize p53 by inhibiting murine double minute 2 (Mdm2),^69^ which leads to tetraploid cell cycle arrest. These data suggest that LATS2 may be involved in CDK4/6i resistance. Consistently, our data further demonstrated that PAX6 promotes CDK4/6i resistance by suppressing LATS2 expression.

Several studies have revealed the relationship between the methylation of the PAX6 promoter and cancer progression^15^; however, whether PAX6 can regulate DNA methylation is largely unknown. The epigenetic factors controlling DNA methylation and histone marks are regulated by PAX6.^48^ Consistently, our data showed that PAX6 can result in LATS2 hypermethylation. A recent study reported that LATS2 hypermethylation is not involved in silencing the mRNA expression of LATS2 mRNA.^70^ However, the absence of LATS2 protein strongly correlates with promoter hypermethylation, where 91 of a total of 107 hypermethylated cases displayed the absence of protein.^71^ Therefore, it is unclear whether LATS2 hypermethylation contributes to decreased LATS2 expression, and whether PAX6 promoting LATS2 hypermethylation is the key mechanism by which PAX6 regulates LATS2 protein expression. Our data showed that PAX6 can regulate epigenetic LATS2 expression by stabilizing the DNMT1 protein, which leads to decreased LATS2 protein expression. However, we cannot fully exclude the possibility that PAX6 expression is suppressed by 5‐Aza, as hypermethylation of PAX6 contributes to various tumor types.^15^, ^72^ Furthermore, whether a feedback loop exists between PAX6, and methylation has not yet been investigated.

The DNA methylation level is a marker of the CDK4/6 inhibitor response.^50^ Treatment with a histone deacetylase inhibitor and a methyltransferase inhibitor resulted in increased levels of p21 and p53 and lower levels of CDK1,^73^ suggesting that methyltransferase inhibitors can affect CDK activity. Furthermore, a recent study indicated that metformin, a widely used anti‐diabetic drug, can mediate tumor suppression in an epigenetic pathway,^74^ and it has been reported that metformin can overcome CDK4/6 inhibitor resistance, and the addition of a methyltransferase inhibitor to CDK4/6i is likely to benefit patients with carcinomas.^75^ In this study, we found that combining 5‐Aza with palbociclib is effective for treating GC, further demonstrating that DNA methylation level can affect CDK4/6 inhibitor response. A recent study suggested that CDK4/6 inhibitors could downregulate DNMT1 expression in Treg cells and trigger anti‐tumor immunity,^76^ suggesting that targeting PAX6 might be of clinical value to tumor immunotherapy in the future.

In conclusion, the present study identified the underlying mechanism of PAX6 inducing CDK4/6 inhibitor resistance in GC, which resulted in the suppression of epigenetic LATS2. Based on our results, we revealed a promising strategy to overcome CDK4/6i resistance by using methyltransferase inhibitors. Our findings may be of great value in the future clinical application of CDK4/6i in treating GC or other malignancies.

## AUTHOR CONTRIBUTIONS

*Study design*: Sen‐lin Zhu and Yi Zhang. *Conducting the experiments*: Yi Zhang, Long‐jun He, Lin‐lin Huang. *Data analysis*: Yi Zhang, Long‐jun He, and Lin‐lin Huang, Nan Lin, Xi‐Wen Wu, Jian‐Liang Xu, Yi Lu, Yan‐Jie Li. *Manuscript writing*: Yi Zhang, Long‐Jun He, Lin‐Lin Huang, Sheng Yao, Ping Li, and Hui‐Wen Xu. All authors read and approved the final manuscript. Yi Zhang, Long‐jun He, and Lin‐lin Huang contributed equally to this work. All authors agreed with the results and conclusions.

## ETHICS APPROVAL AND CONSENT TO PARTICIPATE

This study was approved by the Ethics Committee of The First Affiliated Hospital of Sun Yat‐sen University (Guangzhou, Guangdong, China) and was implemented in accordance with the Helsinki Declaration of Principles.

## CONSENT FOR PUBLICATION

All the authors have agreed to publication of this paper.

## CONFLICT OF INTEREST

The authors declare no conflict of interest.

## AVAILABILITY OF DATA AND MATERIALS

The microarray data used in the study repository were from the TCGA and GEO datasets. The RNA sequencing raw data and normalized results were submitted to the GEO database.

## FUNDING INFORMATION

This work was supported by grants from the National Natural Science Foundation of China (grant number: 81072044, 82002608 and 82002544), the Natural Science Foundation of Guangdong (grant numbers: S2011010004653, 2021A1515011013), Guangzhou Science and Technology Development Funds (grant number: 201803010103), International Cooperation Scientific Research Project of Sun Yat‐sen University in 2018 (grant number: 17), Medical Scientific Research Foundation of Guangdong Province of China (grant number: A2019270), and the Guangdong Basic and Applied Basic Research Foundation (grant number: 2020A1515010178).

## Supporting information

Figure S1 (a) Cell viability of indicated cells following treatment with indicated concentrations of abemaciclib or ribociclib. Chi‐square test: *p < 0.05, **p < 0.01, ***p < 0.001. (b) Co‐IP assay of PAX6 and CCND1 in indicated cells. Three independently repeated experiments were performed with similar results. (c) Representative western blot analysis of p‐Rb (S780) expression in indicated cells with palbociclib, 5‐Aza, DNMT1, or combination of palbociclib, 5‐Aza, and DNMT1. Three independently repeated experiments were performed with similar results Abbreviation: ns, not significant.

Table S1 Correlation between clinicopathological variables and PAX6 expression in gastric cancer
Table S2 Results of univariate analysis for disease‐specific survival in gastric cancer
Table S3 Cox multivariate analyses of prognostic factors on overall survival in gastric cancer
Table S4 Antibody and reagents information
Table S5 Sequences for shRNA
