# Supplementary figures and images for: Oncogenic PAX6 elicits CDK4/6 inhibitor resistance by epigenetically inactivating the LATS2‐Hippo signaling pathway
Source: Clin Transl Med. 2021 Aug 23;11(8):e503. doi: 10.1002/ctm2.503 (PMC8382979; doi:10.1002/ctm2.503)

Supplementary figure 1

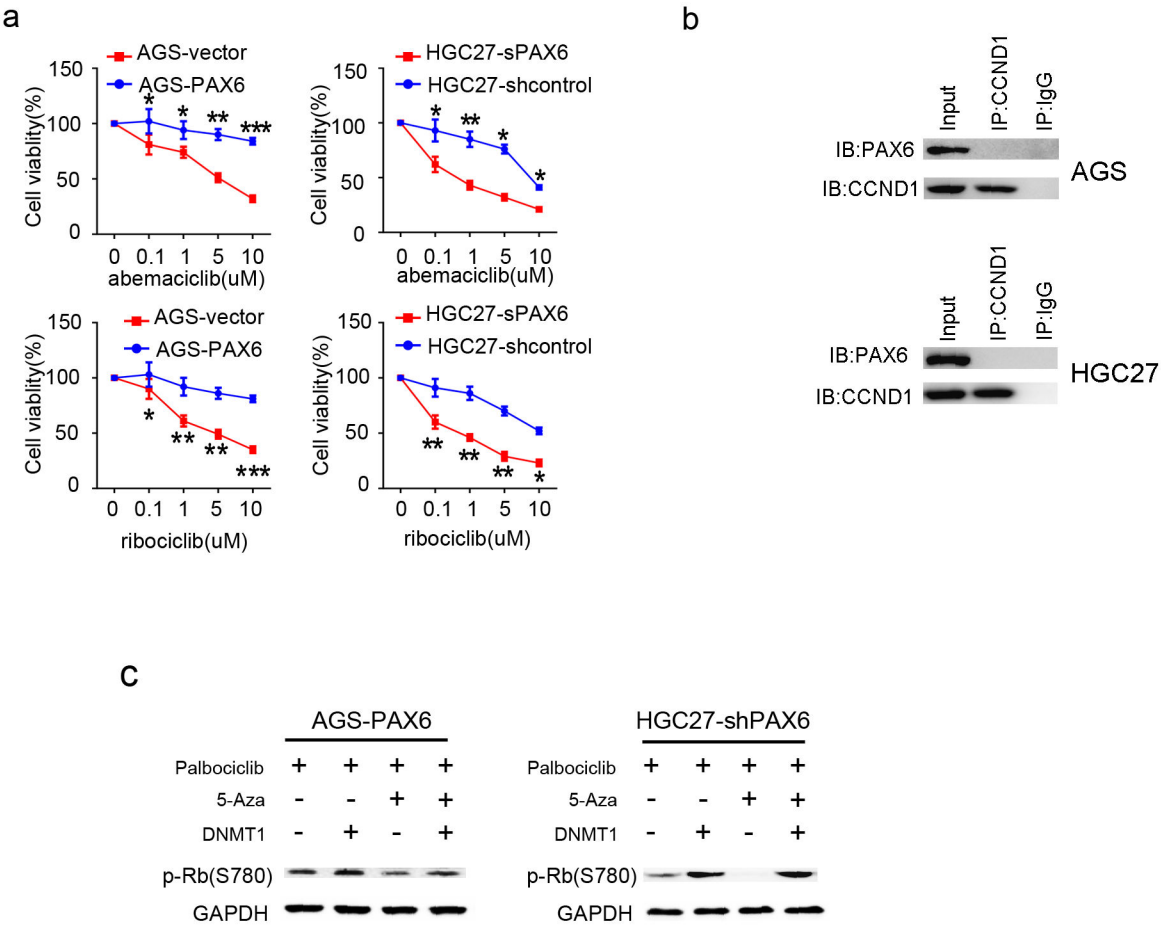

Supplement: Supplementary file 1 — Figure S1 (a) Cell viability of indicated cells following treatment with indicated concentrations of abemaciclib or ribociclib. Chi‐square test: *p < 0.05, **p < 0.01, ***p < 0.001. (b) Co‐IP assay of PAX6 and CCND1 in indicated cells. Three independently repeated experiments were performed with similar results. (c) Representative western blot analysis of p‐Rb (S780) expression in indicated cells with palbociclib, 5‐Aza, DNMT1, or combination of palbociclib, 5‐Aza, and DNMT1. Three independently repeated experiments were performed with similar results Abbreviation: ns, not significant. [file CTM2-11-e503-s001.pdf]
