# Supplementary material for: Oncogenic PAX6 elicits CDK4/6 inhibitor resistance by epigenetically inactivating the LATS2‐Hippo signaling pathway
Source: Clin Transl Med. 2021 Aug 23;11(8):e503. doi: 10.1002/ctm2.503 (PMC8382979; doi:10.1002/ctm2.503)
Supplement: Supplementary file 2 — Table S1 Correlation between clinicopathological variables and PAX6 expression in gastric cancer Table S2 Results of univariate analysis for disease‐specific survival in gastric cancer Table S3 Cox multivariate analyses of prognostic factors on overall survival in gastric cancer Table S4 Antibody and reagents information Table S5 Sequences for shRNA [file CTM2-11-e503-s002.docx]

**Supplementary table 1 Correlation between clinicopathological variables and PAX6 expression in gastric cancer**

| Variable |  | PAX6 protein |  | $x^{2}$ | p value^a^ |
| --- | --- | --- | --- | --- | --- |
|  | All cases | Low expression | High expression |  |  |
| **Age (years)^b^** |  |  |  | 0.094 | 0.759 |
| <60 | 174 | 59 | 115 |  |  |
| ≥60 | 44 | 16 | 28 |  |  |
| **Gender** |  |  |  | 1.123 | 0.289 |
| Female | 34 | 9 | 25 |  |  |
| Male | 184 | 66 | 118 |  |  |
| **Tumor size (cm)** |  |  |  | 7.016 | **0.008** |
| <5 | 76 | 35 | 41 |  |  |
| ≥5 | 142 | 40 | 102 |  |  |
| **Depth of invasion** |  |  |  | 11.009 | **0.001** |
| T1/T2 | 100 | 46 | 54 |  |  |
| T3/T4 | 118 | 29 | 89 |  |  |
| **Differentiation** |  |  |  | 9.652 | **0.003** |
| Well-Moderate | 123 | 45 | 78 |  |  |
| Poor-Undifferentiated | 95 | 30 | 65 |  |  |
| **Stage** |  |  |  | 13.414 | **0.000** |
| I-II | 108 | 50 | 58 |  |  |
| III-IV | 110 | 25 | 85 |  |  |
| **Lymph node Metastasis** |  |  |  | 10.181 | **0.001** |
| Yes | 111 | 27 | 84 |  |  |
| No | 107 | 48 | 59 |  |  |

**Supplementary table 2 Results of univariate analysis for disease-specific survival in gastric cancer**

| Variable | Subvariable | All cases (n=218) | | |
| --- | --- | --- | --- | --- |
|  |  | HR | 95% CI | P value |
| Univariate |  |  |  |  |
| Age | ≥60 yr (n = 174) vs <60 yr (n = 44) | 0.995 | 0.979–1.011 | 0.560 |
| Gender | Female (n = 34) vs Male (n = 184) | 1.002 | 0.577–1.738 | 0.995 |
| Tumor Size | ≥5 (n = 142) vs <5 (n = 76) | 1.833 | 1.228–2.875 | **0.005** |
| Differentiation | Well-Moderate (n = 123) vs Poor-Undifferentiation (n = 95) | 1.969 | 1.322–2.934 | **0.001** |
| Depth of invasion | T3+T4 (n = 118) vs T1+T2 (n = 100) | 2.507 | 1.634-3.846 | **0.000** |
| Stage | III-IV (n = 117) vs I-II (n = 101) | 1.843 | 1.239–2.742 | **0.003** |
| Metastasis | Yes (n = 120) vs No (n = 98) | 4.593 | 2.865–7.361 | **0.000** |
| PAX6 expression | Low expression (n = 145) vs High expression (n = 73) | 4.160 | 2.364–7.322 | **0.000** |

Supplementary table 3 Cox multivariate analyses of prognostic factors on overall survival in gastric cancer

| Variable | β | SE | Hazard ratio | 95%CI | p value |
| --- | --- | --- | --- | --- | --- |
| Depth of invasion | 0.763 | 0.2222 | 2.145 | 1.388-3.315 | **0.001** |
| Tumor size | / | / |  | / | / |
| Stage (I–II vs III–IV) | 0.505 | 0.232 | 1.657 | 1.052-2.610 | **0.029** |
| Differentiation | 0.450 | 0.223 | 1.568 | 1.013-2.428 | **0.044** |
| Metastasis | 1.328 | 0.259 | 3.774 | 2.272-6.269 | **0.000** |
| PAX6 | 0.933 | 0.300 | 2.542 | 1.411-4.579 | **0.002** |

**Supplementary table 4. Antibody and reagents information**

| **Antibody name** | **Source** |
| --- | --- |
| PAX6 | Santa Cruz (sc-81649) |
| GAPDH | Proteintech (10494-1-AP) |
| Cyclin D1 (CCND1) | Cell Signal (55506) |
| Phospho-Rb (Ser780) | Cell Signal (8180) |
| Phospho-Rb (Ser795) | Cell Signal (9301) |
| Rb | Cell signal (9309) |
| E2F1 | Cell Signal (3742) |
| CDK6 | Cell Signal (13331) |
| Cyclin D2 (CCND2) | Cell Signal (3741) |
| Cyclin D3 (CCND3) | Cell Signal (2936) |
| Cyclin E | Cell Signal (4129) |
| CDK1 | Abcam (ab32094) |
| CDK9 | Abcam (ab76320) |
| LATS1 | Cell Signal (3477) |
| LATS2 | Cell Signal (5888) |
| MST1 | Cell Signal (3682) |
| YAP | Cell Signal (4912) |
| Phospho-YAP (Ser127) | Cell Signal (13008) |
| TAZ | Cell Signal (83669) |
| Phospho-TAZ (Ser89) | Cell Signal (59971) |
| WWC1 (KIBRA) | Cell Signal (8774) |
| LAMIN B1 | Abcam (16048) |
| PARP | Cell Signal (9532) |
| Cleaved PARP | Cell Signal (5625) |
| Caspase-3 | Cell Signal (9662) |
| Cleaved Caspase-3 | Cell Signal (9664) |
| DNMT1 | Cell Signal (5032) |
| DNMT3a | Cell Signal (32578) |
| DNMT3b | Cell Signal (57868) |
| **Drugs name** | **Source** |
| 5-Aza-2'-deoxycytidine (Decitabine) | Abcam (ab120842) |
| Palbociclib | Cell signal (47284) |
| Abemaciclib | Selleck chemicals (S5716) |
| Ribociclib | Selleck chemicals (S7440) |

**Supplementary table 5. Sequences for shRNA**

| Name | Sequence |
| --- | --- |
| PAX6-RNAi-1 | GCGACTCCAGAAGTTGTAA |
| PAX6-RNAi-2 | GCAGACGGCATGTATGATA |
| PAX6-RNAi-3 | GCTTCACCATGGCAAATAA |
| CCND1-RNAi-1 | AAATCACAAAGTCCTGCAT |
| CCND1-RNAi-2 | GGATGAACCAGGAAGATTT |
| CCND1-RNAi-3 | CCTGCATTACGGAGACTTA |
| LATS2-RNAi-1 | GCACGCAUUUUACGCCUUA |
| LATS2-RNAi-2 | ACACUCACCUCGCCCAAUA |
| LATS2-RNAi-3 | GAAGUGAACCGGCAAAUGC |
| CCND2-RNAi-1 | AGCCAGTATATACGACACT |
| CCND2-RNAi-2 | ACGAGGTAATGTCCAACAT |
| CCND2-RNAi-3 | CCGTATGCAGCACAAGAAA |
